# Serum Interleukin-18 Expression in Children With Bronchial Asthma

**DOI:** 10.1097/WOX.0b013e3181a33649

**Published:** 2009-05-15

**Authors:** Elham M Hossny, Shereen S El-Sayed, Eman S El-Hadidi, Sherif R Moussa

**Affiliations:** 1 Department of Pediatrics, Cairo, Egypt; 2 Department of Clinical Pathology, Ain Shams University, Cairo, Egypt

**Keywords:** IL-18, asthma, children, inflammation, exacerbation, pathogenesis

## Abstract

**Background**

Interleukin 18 (IL-18) is reported to have regulatory functions on Th1 and Th2 cytokine production and proinflammatory effects through promoting recruitment of memory Th1 cells to inflammatory sites. We sought to investigate the expression of the serum IL-18 in childhood bronchial asthma in relation to disease activity and severity.

**Methods**

Serum IL-18 was measured by enzymatic immunoassay in 25 asthmatic children during exacerbation and after complete quiescence of symptoms and signs. The results were compared to those of 35 nonallergic age- and sex-matched children.

**Results**

Serum IL-18 levels during asthma exacerbation [median = 125 pg/mL; mean (SD) = 128.6 (43.3) pg/mL] were significantly lower than the follow-up levels during stability [median = 250 pg/mL; mean (SD) = 291.6 (66.7) pg/mL] and both levels correlated positively with each other. The corresponding values of the control group were higher than those of the asthmatic patients whether during exacerbation or stability [median = 380 pg/mL; mean (SD) = 476.1 (259.6) pg/mL]. The serum IL-18 concentrations did not vary significantly according to asthma severity, family history of atopy, or passive smoking. The influence of inhaled corticosteroids on IL-18 expression was not impressive and neither was the relation between serum IL-18 and the peripheral blood eosinophil count or serum total IgE expression.

**Conclusions**

Serum IL-18 was found underexpressed in a group of asthmatic children especially during exacerbation. Further studies are needed to outline its exact role in the pathogenesis of asthma.

## 

Interleukin-18 is a pleiotrophic cytokine involved in both Th1 and Th2 differentiation [1]. It is predominantly produced by activated macrophages and synergizes with IL-12 and IL-2 to induce IFN-*γ *synthesis, thereby promoting Th1 cytokine response [2,3]. It was hypothesized that reduced IFN-*γ *secretion in the presence of increased secretion of IL-18 may imply a defect in IL-18 responsiveness in allergic patients [4]. It was reported that a deficiency of IFN-*γ *production in severe atopic dermatitis (AD) patients could not be reversed by IL-18 in vitro [5].

IL-18 may stimulate Th1 cells in vivo to produce also IL-13, granulocyte-macrophage colony stimulating factor, and IL-8, which in combination might induce severe inflammation [6]. IL-18 has chemotactic activities in vitro and in vivo operating in particular upon human Thl cells. Thus, in addition to its activities in functional regulation of naive T cell differentiation, it may have an important role in promoting recruitment of memory Thl cells to an inflammatory lesion [7]. It may possess some other biologic properties including upregulation of intercellular adhesion molecule-1 and Fas ligand in human myelomonocytic cells [8,9].

Paradoxically, IL-18, by itself, strongly induces IgE and allergic inflammation, indicating a role for IL-18 in promoting Th2 response [3]. Although IL-18 was initially thought to negatively regulate CD4^+ ^cell-derived IL-4 synthesis, via an IFN-*γ *independent mechanism, thereby limiting the development of type 2 immune responses,[10] later studies revealed its potential to induce Th2 cytokine production from T cells, NK cells, and basophils/mast cells [6]. It seems that IL-18 has the ability to promote both Th1 and Th2 responses, depending on the surrounding cytokine environment [11].

IL-18 was found to induce allergic inflammation in a mouse model and its role in AD in particular has been outlined. Depletion of IL-18 was found to rescue the mice from AD, indicating IL-18-dependent, Th2-independent AD. This type of AD was classified as innate-type allergy in contrast to Th2 cell-dependent ordinary allergy. It may be categorized as Th1-associated allergy and it would be interesting to evaluate whether individual allergic disorders involve either of these IL-18-mediated pathways or a Th2- mediated one [12]. The functional role of IL-18-induced "innate allergic response" was also explained in the context of host defense against gastrointestinal nematode infection by activating intestinal mucosal mast cells in a Th2-independent manner [13].

This background stimulated our study of the IL-18 expression in the serum of children with bronchial asthma during and after subsidence of exacerbations in relation to severity and other disease traits.

## Methods

### Study population

This follow-up study comprised 25 asthmatic and 35 healthy children. The asthmatic children were enrolled consecutively from the Pediatric Allergy and Immunology Unit of the Children's Hospital in Cairo, Egypt during an acute asthma exacerbation. The diagnosis of asthma was established according to the American Thoracic Society criteria [14]. There were 16 boys and 9 girls. Their ages ranged from 2.5 to 11.5 years with a median age of 8 (mean = 8 ± 3 years). The patients were classified according to severity of exacerbation into 5 children with mild, 5 with moderate, and 15 with severe exacerbation and were classified during stability into 7 patients with mild persistent, 14 with moderate persistent, and 4 with severe persistent asthma [15]. At time of initial sampling, 17 children (68%) were compliant on regular corticosteroid inhalation therapy (fluticasone propionate 100-250 *μ*g/day) whereas 8 children (32%) were not receiving corticosteroids. Their chest radiographs were normal and cases with concomitant illness were excluded from the study. The patients were subjected to the study measurements at enrollment and were followed up to be re-evaluated after clinical subsidence of symptoms and signs.

The control group comprised 35 healthy children (22 boys and 13 girls). Their ages ranged from 2 to 10.5 years (median = 6.5 years; mean = 6.5 ± 2.5 years). They had no present, past, or family history suggestive of allergy. An informed consent was obtained from the parents or caregivers of each subject before enrollment and the study protocol gained approval from the ethics committee of the Department of Pediatrics, Ain Shams University.

### Study measurements

Five milliliters of blood was aseptically collected in aliquots by venepuncture from each subject in sterile tubes for blood cell counting and serum Il-18 and total IgE assays. The serum was separated by centrifugation at 1500 rpm for 15 minutes after 2 hours of incubation at 25°C and was stored at - 20°C until assayed.

#### Serum IL-18 level

The serum IL-18 level was quantitatively estimated using a human IL-18 immunoassay (Bender MedSystems GmbH, Campus Vienna Biocenter 2, A-1030 Vienna, Austria) according to the manufacturer's instructions. The assay employs the quantitative sandwich enzyme immunoassay technique.

#### Serum total IgE

The serum total IgE was measured by ELISA (Genzyme Diagnostics, Medix Biotech, San Carlos, Calif). A serum IgE level was considered elevated if it exceeded the highest reference value for age [16]. The value used in correlation analysis was the percentage from the highest normal for age (patient's actual level divided by the highest reference value for age multiplied by 100). Subjects in the control group with elevated serum total IgE levels were excluded from the study.

#### Complete blood counting

Complete blood counting was performed with use of a cell counter (Coulter MicroDiff 18, Fullerton, Calif) and by manual differential. Blood sampling of all subjects was performed at the same time daily (10 AM) to avoid diurnal variation of eosinophil count.

### Statistical analysis

The results were statistically analyzed via a standard computer program (SPSS version 10 for Windows, Chicago, Ill). The data are expressed as median, interquartile range, mean, and SD. For analysis of the nonparametric data, Mann-Whitney *U *test, Wilcoxon signed rank test, and Kruskal-Wallis tests were used. A multivariate analysis employing multiple linear regression was used to relate the magnitude of the drop in serum IL-18 ("delta") to other data of the patients using the analysis of variance test. Correlations were evaluated by the Spearman's rank correlation coefficient. For all tests, *P *values less than 0.05 were considered significant.

## Results

**Figure 1:**
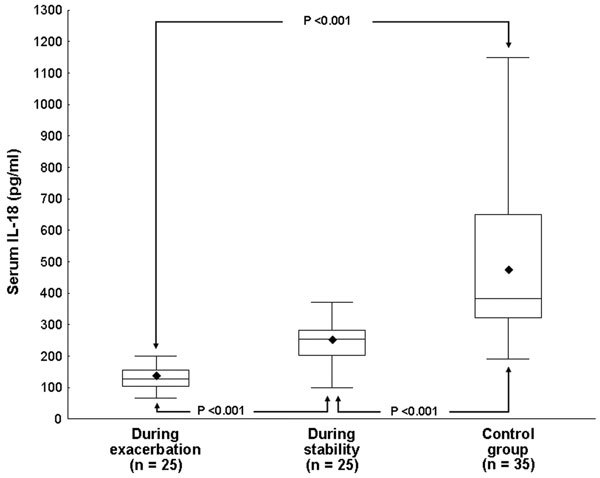


**Figure 2:**
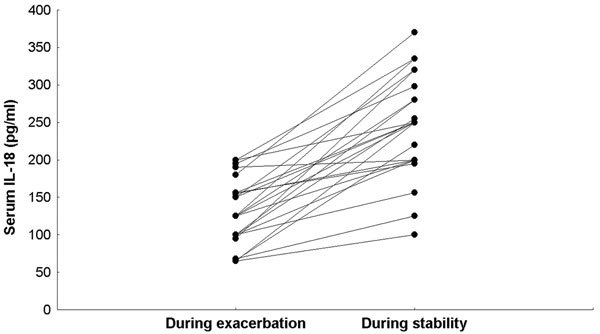


Serum IL-18 levels during asthma exacerbations ranged from 65 to 200 pg/mL [median = 125 pg/mL; mean (SD) = 128.6 (43.3) pg/mL]. These values were significantly lower than those of the same patients when studied after subsidence of symptoms and signs [range = 100-370 pg/mL; median = 250 pg/mL; mean (SD) = 241.6 (66.7) pg/mL]. The healthy children had significantly higher serum IL-18 levels [range = 190-1150 pg/mL; median = 380 pg/mL; mean (SD) = 476.1 (259.6) pg/mL] compared with the patients' levels whether during exacerbation or stability (Figures 1, 2).

The serum IL-18 expression did not vary significantly with the severity of exacerbation. Levels of patients with mild attacks ranged from 66 to 200 pg/mL [median = 100 pg/mL; mean (SD) = 124.2 (53.0) pg/mL]. The corresponding levels during moderate and severe attacks ranged from 68 to 195 pg/mL [median = 100 pg/mL; mean (SD) = 121.6 (50.6) pg/mL] and from 65 to 200 pg/mL [median = 125 pg/mL; mean (SD) = 132.5 (40.6) pg/mL], respectively. The difference was statistically insignificant (*P *= 0.780). Similarly, IL-18 levels were comparable between patients with variable severity grades during stability. Children with mild persistent asthma had levels that ranged from 156 to 320 pg/mL [median = 250 pg/mL; mean (SD) = 24.9 (65.7) pg/mL]. The corresponding levels of children with moderate persistent and severe persistent asthma ranged between 125 and 370 pg/mL [median = 250 pg/mL; mean (SD) = 247.5 (60.4) pg/mL] and between 100 and 335 pg/mL [median = 197.5 pg/mL; mean (SD) = 207.5 (96.7) pg/mL], respectively (*P *= 0.485).

The patients' IL-18 levels were not influenced by the family history of atopy, inhalation corticosteroid therapy, or serum total IgE expression whether during asthma exacerbation or during stability. It is worth noting that only 6 (24%) asthmatic children in our series had normal serum total IgE concentration. Out of the 25 asthmatic children studied, 17 were considered passive smokers as a result of their exposure to tobacco smoke from household contact. Their serum IL-18 expression was comparable to that of the 8 asthmatic children who did not report exposure to tobacco smoke inside their homes. The same observation was made among the children in the control group, of whom 21 out of 35 were passive smokers (Table 1). The serum IL-18 expression did not correlate with the age, weight, and height percentiles, duration of asthma, hemoglobin concentration, or total leukocyte, eosinophil, neutrophil, lymphocyte, and monocyte counts of the patients.

**Table 1:** Variation of Serum IL-18 Expression According to Some Variables

	Serum IL-18 [Median (IQR) pg/ml]	
	
Variables	During Exacerbation	During Stability
Serum Total IgE		
Elevated	125 (56)	250 (80)
Normal	140 (91.75)	252.5 (134.75)
z	0.578	0.483
p	0.598	0.642
Family History of Allergy		
Positive	125 (56)	250 (109)
Negative	112.5 (97.75)	235 (80)
z	0.521	0.869
p	0.611	0.894
Inhalation Corticosteroid Therapy		
Positive	125 (62)	250 (75)
Negative	100 (60)	250 (98)
z	0.663	0.111
p	0.536	0.936
Passive Smoking		
Positive	100 (71)	250 (67.5)
Negative	153 (62.5)	265 (114.5)
z	1.88	0.825
p	0.066	0.44

The drop in IL-18 values (*δ*) between exacerbation and remission could not be correlated by Spearman correlation coefficient to age (*P *= 0.58), serum total IgE (*P *= 0.87), duration of exacerbation (*P *= 0.94), absolute eosinophil count (*P *= 0.29), or other blood cell counts. Also, a multiple linear regression analysis could not relate the *δ *values to the serum total IgE (*P *= 0.49), passive smoking (*P *= 0.78), duration into exacerbation (*P *= 0.08), or blood cell counts. However, the drop in IL-18 was positively related to the age of the patients by regression analysis (*P *= 0.027). The severity of exacerbation did not significantly affect the magnitude of drop when evaluated by Kruskal-Wallis multivariate analysis (3.425; *P *= 0.18).

The duration since onset of exacerbation at the time of initial sampling ranged between 4 and 26 hours [median = 10 hours; mean (SD) = 11.2 (6.2) hours]. The duration of exacerbation was negatively correlated to the eosinophil count of patients (r = -0.53; *P *= 0.007) but to none of the other studied clinical or laboratory parameters.

## Discussion

Serum IL-18 was underexpressed in a group of children with bronchial asthma in comparison with that of a matched group of normal children. After subsidence of exacerbation, serum IL-18 levels increased significantly but were still lower than the control values. This comes in accordance with Cebeci et al [17] who reported significantly low serum IL-18 levels in children with acute asthma (median = 8.55 pg/mL) compared with those of a group of controls (median = 140.10 pg/mL). Another investigation that sought to evaluate its expression in bronchoalveolar lavage fluid (BAL) and supernatant of lipopolysaccharide-stimulated alveolar macrophages in adults with bronchial asthma revealed a marked decrease of IL-18 and suggested that inherently low levels of IL-18 may be associated with the pathogenesis of asthmatic airway inflammation. IL-18 may be implicated at an early stage of the pathogenesis of the disease, and the downstream effect of this deficiency interacts with other mediators to modulate disease severity [18]. There is evidence that the role of IL-18 in development of a critical Th1 response occurs early in this response [19]. IL-18 might hypothetically be consumed in the process of allergic inflammation. Cameron et al [20] demonstrated that IL-18 is constitutively expressed in the airway epithelium and parenchyma of murine lung tissue and human bronchial biopsies and that its expression is increased under conditions characterized by Th1 cytokine expression such as lipopolysaccharide stimulation and pulmonary sarcoidosis and reduced during Th2 cytokine-mediated conditions such as ovalbumin challenge and asthma.

In contrast, a study of 370 schoolchildren aged 9 to 10 years living in urban Japanese areas revealed that serum IL-18 levels were significantly higher in children who had asthma, allergic rhinitis, or atopic eczema than in the rest of the enrolled children [21]. The same was observed during asthma exacerbation and convalescence in 34 Chinese children [22]. Another investigation observed that plasma IL-18, IL-I2, IL-10, and IL-13 levels were significantly higher in allergic asthmatic patients than in normal control subjects [23]. They explained the excessive IL-18 production by the elevation of IL-12 levels. El-Mezzein et al [4] found no significant difference in plasma IL-18 concentration between a group of adult asthmatics and nonallergic controls despite its elevation in the supernatant fluid of lipopolysaccharide-stimulated peripheral blood mononuclear cells (PBMC) in the former group. However, the spontaneous secretion of IL-18 by unstimulated PBMC was very low or immeasurable, and mature form IL-18 was not present in lysates of unstimulated PBMC from asthmatic patients and nonallergic controls. IL-18 concentration was recently reported to be increased in the BAL fluid, but not in the nasal lavage fluid, in a mouse model of allergic rhinitis [24].

In the current study, a positive correlation was observed between serum IL-18 values during and after subsidence of exacerbation--meaning that the lower the IL-18 became during exacerbation, the lower it remained after quiescence. This observation probably reflects the persistence of airway inflammation during stability. Serum IL-18 expression or magnitude of drop at quiescence was not influenced by sex, or body size as determined by weight and height percentile values. The degree of drop (*δ*) was positively related to age. The finding is however limited by the sample size.

Serum IL-18 expression did not vary with the degree of asthma severity whether during exacerbation or stability. Also, the degree of drop in IL-18 between exacerbation and remission did not correlate with exacerbation severity. It seems that IL-18 expression is related to the airway inflammatory processes in persistent bronchial asthma irrespective of clinical severity and it is well known that even patients with mild persistent asthma may have ongoing allergic inflammation in their airways throughout the disease course including the periods of quiescence of symptoms.

Passive smoking did not influence the serum IL-18 expression or magnitude of drop in our series. However, most children belonged to the passive smoker category, which might have influenced this observation. McKay et al [25] reported that cigarette smokers had significantly reduced sputum IL-18 mRNA expression compared with nonsmokers. This effect was more pronounced in asthmatics than in normal subjects. Such observation may partly offer an explanation for the reduced IL-18 expression in our investigation given the high levels of environmental tobacco smoke exposure in some developing countries including Egypt.

Serum IL-18 levels in our series did not vary with the peripheral blood eosinophil count. A relevant study reported that IL-18 and IL-12 inhibit antigen-induced airway hyper-responsiveness, lung eosinophilia, and serum IgE, but IL-18 or IL-12 alone failed to modulate any of these allergic responses [26]. However, several investigations showed that in vivo IL-18 administration alone enhanced allergen-induced eosinophilic recruitment to the lungs, Th2 cytokine, and IgE production in sensitized mice [27,28]. IL-18 deficiency thus appears to promote allergic inflammation characterized by eosinophilia [26,29]. The latter data, however, are not unchallenged [18]. Hence, IL-18 is likely to have different functions, possibly dependent on where and when it is produced [30]. The combination of IL-2 and IL-18 was recently found to prevent airway hyperresponsiveness and airway inflammation in a mouse model, likely through IL-12-mediated induction of IFN-*γ *production in NK cells [31]. Intraperitoneal administration of recombinant IL-18 reduced these antigen-induced changes in association with a decrease in IL-4 in BAL fluid and lung tissue. However, it did not affect the IFN-*γ *level and somewhat enhanced the production of IL-5 [29].

Serum IL-18 expression or degree of drop between exacerbation and quiescence of symptoms and signs could not be related to the family history of atopy or serum total IgE concentration. It is possible that the change in IL-18 expression reflects the inflammatory process regardless of the atopy status or IgE levels. Accordingly, a recent study indicated that serum IL-18 levels were associated with allergic symptoms in children, independent of serum IgE levels [21]. Also, sputum IL-18 did not correlate with serum IgE levels in a study of adult asthmatics [25]. However, in vitro IL-18 secretion by lipopolysaccharide-stimulated PBMC was positively correlated to the plasma IgE concentration [4]. Administration of IL-18 alone significantly increased the IgE level production in vivo by stimulated PBMC in nonsensitized mice, and the administration of IL-18 and IL-12 induced a 70-fold higher serum level of IgE [32]. The contradiction needs further wider scale studies for verification.

We did not find an influence for the low-dose inhaled corticosteroids (ICS) used before enrollment (100-250 *μ*g of fluticasone propionate per day) on serum IL-18 expression. Increased IL-18 concentrations decreased after steroid treatment in a mouse model [24]. A study on humans reported that patients with bronchial asthma receiving ICS had reduced levels of IL-18 in BAL fluid irrespective of the dose they were receiving. The authors, however, stated that the effect of corticosteroids on IL-18 is currently uncertain because the sample with the highest level of IL-18 in their series came from a patient with asthma who was receiving 7 mg of prednisolone continuously (in addition to 800 *μ*g/day of inhaled budesonide). In that instance, high doses of inhaled and systemic corticosteroid did not cause any hypothetical corticosteroid suppression of IL-18 synthesis [18].

The time elapsed from the start of exacerbation untill initial sampling did not influence the serum IL-18 expression or magnitude of drop at the time of second sampling. However, a weak negative correlation could link the duration of exacerbation to the eosinophil count at enrollment. None of the other clinical or laboratory data of the patients were significantly related to the spread of time into exacerbation within the study group.

Manipulation of IL-18 levels may prove of value in many allergic and nonallergic disorders through modulation of inflammation, but its influence on other mediators such as IFN-*γ *should be kept in mind before conducting clinical trials on experimental animals or human beings. It was suggested that antioxidants may reduce IL-18 expression in asthma by inhibiting the activity of NF-*Κ*b [11].

The study has certain limitations for credibility. First, the sample size is too small for solid conclusions to be formed. Second, for one to objectively study the effect of ICS on serum IL-18 expression, a prospective study design is needed in which a group of steroid-naive subjects are tested before and after a reasonable period of regular ICS therapy. Finally, the heterogeneity of the patient population may place constraints on the conclusions.

From our data, it seems that serum IL-18 is underexpressed in childhood asthma probably because of consumption at an early stage of asthma pathogenesis. Wider scale studies are indicated to verify the alterations in IL-18 levels according to the different protocols of asthma therapy. Given the complexity of the mechanisms of IL-18 actions in the airway, further evaluation of its exact biologic significance in the asthmatic inflammatory process is needed.
